# Development and validation of an early predictive model for acute stress disorder after trauma: a clinical cohort study

**DOI:** 10.7717/peerj.21131

**Published:** 2026-04-13

**Authors:** Xiahong Li, Shangpeng Shi, Juan Gu, Xiangyuan Chu, Xiu Dai, Xiuquan Shi

**Affiliations:** 1 Medical Reform Office, The Third Affiliated Hospital of Zunyi Medical University, The First People’s Hospital of Zunyi, Zunyi, Guizhou, China; 2 Zunyi Medical and Pharmaceutical College, Zunyi, Guizhou, China; 3 Department of Epidemiology and Health Statistics, School of Public Health, Zunyi Medical University, Zunyi, Guizhou, China; 4 Key Laboratory of Maternal & Child Health and Exposure Science of Guizhou Higher Education Institutes, Zunyi, Guizhou, China; 5 Center for Pediatric Trauma Research & Center for Injury Research and Policy, The Abigail Wexner Research Institute at Nationwide Children’s Hospital, The Ohio State University College of Medicine, Columbus, OH, United States of America

**Keywords:** Trauma, ASD, Epidemiological characteristics, Inflammation, Predictive model

## Abstract

**Background:**

Injury is sudden and unpredictable and has become a major public health problem in the world, and many trauma patients may experience cognitive or psychological problems including acute stress disorder (ASD). However, the ability to identify ASD early is still limited. The aim of this study was to investigate the risk factors for post-traumatic ASD and to establish a visual prediction model.

**Methods:**

This prospective cohort study was conducted from the trauma center of one general hospital in Zunyi. Cases of 216 inpatients with trauma were selected from September 2020 to August 2021. The participants were divided into the ASD (*n* = 49) and non-ASD (*n* = 167) groups according to the diagnostic criteria for ASD. General demographic characteristics and clinical information were collected. To establish a prediction model, the Least Absolute Shrinkage and Selection Operator (LASSO) regression method was applied to filter variables, and multivariable logistic regression analysis was used to construct a nomogram. The nomogram performance was determined by its discrimination, calibration, and clinical usefulness.

**Results:**

Patients in the ASD group differed significantly from those in the non-ASD group in terms of general demographic and clinical characteristics (*e.g.*, work or life pressure, trauma history, cause of trauma, trauma site, coma, fear, psychological burden, critical condition, sleep quality, limb activity). The ASD group exhibited higher levels compared to the control group in inflammatory markers, which indicated that ASD might be associated with inflammation in trauma patients. The predictive model yielded an Area Under the Curve (AUC) of 0.846 (95% Confidence Interval (CI) [0.781–0.911]), sensitivity was 67.35%, specificity was 91.62%, and in the internal validation, the AUC was 0.845 (95% CI [0.783–0.911]). This model showed good calibration and positive net benefits in decision curve analysis when the risk threshold of ASD was between 10% and 83%.

**Conclusions:**

Our prediction model had a good discriminatory capacity and showed better effects in calibration. It may have potential value for the early identification of ASD.

## Introduction

Injury is one of the leading causes of death and disability worldwide, claiming approximately 4.4 million lives each year and accounting for 8% of all deaths (World Health Organization, 2024), thereby constituting a significant cause of global mortality and morbidity (Rapsang & Shyam, 2015). With the incessant advancement of medical therapy, physical trauma may be assuaged or even remedied entirely. However, the conspicuous upsurge in the occurrence of mental disorders ensuing from the ordeal of experiencing trauma remains a burgeoning concern (Howlett, Nelson & Stein, 2022; Worku, Tesfaw & Getnet, 2022). A Canadian cohort study found that women with traumatic brain injury (TBI) had an 18.5% risk of being diagnosed with a new psychiatric disorder during the perinatal period (Brown et al., 2025). An analysis of 2,163 adults with TBI from Europe and Israel reported that 7–18% screened positive for probable Major Depressive Disorder (MDD) and approximately 10% for probable Post-Traumatic Stress Disorder (PTSD) (Mikolić et al., 2025). In America, a prospective study on adult survivors of major burn injuries reported that the incidence of PTSD at 1, 6, 12 and 24 months post-injury was 35.1%, 33.3%, 28.6%, and 25.4%, respectively (McKibben et al., 2008). A longitudinal study from Australia found that 54% of trauma survivors reported above normal scores for depression, anxiety and/or stress in at least one of three interviews conducted within the 6-month follow-up period (Wiseman et al., 2015).

Acute stress disorder (ASD) was initially conceptualized in the Diagnostic and Statistical Manual of Mental Disorders, version 4 (DSM-4) in the United States in 1994 (American Psychiatric Association, 1994) and it is a short-term acute stress reaction that occurs within two days to a month after an individual experiences death, fear, or other traumatic events (Bryant et al., 1998). ASD is characterized by four symptom clusters: psychic dissociation, recurrent re-experiencing of the traumatic event, avoidance, and hypervigilance (Bryant et al., 1998). A study conducted in Northwest Ethiopia revealed a prevalence of probable ASD at 45% (95% Confidence Interval, (CI) [40.2–49.6]) (Worku, Tesfaw & Getnet, 2022). In a nested case-control study involving 175 hospitalized burn patients, 41 were diagnosed with ASD, resulting in an incidence of 23.4% (Tamayo-Gómez et al., 2022). Moreover, a review study reported ASD prevalence during hospitalization ranging from 1% to 37% (Visser et al., 2017). Several studies have noted that ASD shares similar symptom clusters with PTSD and serves as a significant predictor of PTSD (Armour & Hansen, 2015; Astill Wright et al., 2019). Bryant (2018) and colleagues observed that chronic PTSD manifested in approximately two-thirds of ASD patients experiencing post-traumatic stress. Additionally, in a similar study, it was also demonstrated that ASD occurred in 19% of victims of violent assault, with 83% developing PTSD six months post-assault (Brewin et al., 1999). The main difference between ASD and PTSD lies in the former’s emphasis on dissociative reactions to the trauma and the duration of the symptoms (Bryant & Harvey, 2003). Specifically, a diagnosis of ASD requires the individual to experience at least three dissociative symptoms and ASD can be diagnosed between two days and four weeks after the trauma, whereas, PTSD can be diagnosed only after a minimum of four weeks post-trauma (Dai et al., 2018). In summary, ASD serves as a valuable reference for predicting PTSD (Armour & Hansen, 2015; Astill Wright et al., 2019); early and timely intervention in high-risk populations is therefore essential to prevent the progression from ASD to chronic PTSD (Astill Wright et al., 2019).

Studies have shown that ASD is common among adults exposed to traumatic events (Briere et al., 2017; Hagos et al., 2024), and younger patients are at greater risk than those middle-aged or older (Liang et al., 2022). Women were more likely to develop psychological symptoms after stressful incidents (Parker et al., 2021). Previous work showed that a history of trauma, complications, and injury severity could compromise vital functions of the body, eroding self-care, independence and social engagement, thereby intensifying ASD (Melcer et al., 2019; Mona, Chimbari & Hongoro, 2019). Prolonged hospitalization was significantly associated with ASD (Nosanov et al., 2022) and delayed discharge might indicate clinical deterioration and further increase the likelihood of developing ASD (Hagos et al., 2024). Patients with intense pain perception also faced higher ASD risk (Liang et al., 2022). Previous studies have pointed out that pain could be used as a source of stress to cause patients to evoke additional negative emotions (Biçen et al., 2021; Johnson et al., 2020).

ASD is presently diagnosed chiefly through developmental history, structured behavioral observation and caregiver/self-report. However, objective and precise biological predictive indicators remain scarce. Accumulating evidence indicates that immune–inflammatory dysregulation contributes to the pathophysiology of mood disorders (Dionisie et al., 2021; Rosenblat & McIntyre, 2017). Adults with post-traumatic stress disorder exhibit higher circulating levels of C-reactive protein (CRP), Interleukin-6 (IL-6) and Tumor Necrosis Factor-α (TNF-α) than control subjects (Peruzzolo et al., 2022). Furthermore, a review study on depression indicated that increased expression of interferons, Interleukin-1beta (IL-1β), TNF-α, and other pro-inflammatory cytokines could disrupt neurotransmitter metabolism and neuroplasticity through interference with serotonin synthesis, release of toxic metabolites, and induction of astrocytes to release reactive oxygen species and active nitrogen (Grolli et al., 2021; Miller & Raison, 2016). At present, there remains a dearth of evidence concerning the association between inflammation and post-traumatic ASD.

In recent years, the ratios of various leukocyte subtypes, such as the Neutrophil-to-Lymphocyte Ratio (NLR), Platelet-to-Lymphocyte Ratio (PLR), and Monocyte-to-Lymphocyte Ratio (MLR) have emerged as recognized biomarkers for assessing the overall inflammatory status and are deemed superior indicators of pro-inflammatory activity (Hu et al., 2021; Jung et al., 2019; Wang et al., 2020). NLR has garnered recognition as a novel marker for chronic low-grade inflammation and a predictor of clinical prognosis in neuroimmune diseases (Akıl et al., 2015; Ivković et al., 2016). Presently, NLR, PLR, and MLR have been investigated in psychiatric disorders. A meta-analysis revealed higher NLR and PLR in individuals with Bipolar Disorder (BD) compared to healthy controls (Mazza et al., 2018). Additionally, elevated MLR levels were observed in patients with schizophrenia, BD, and depression (Dionisie et al., 2021; Özdin, Sarisoy & Böke, 2017). The Systemic Immune-Inflammation Index (SII), a novel inflammatory biomarker derived from a combination of NLR and PLR (Dionisie et al., 2021), has been widely applied to cardiovascular disease, infection, inflammatory disease, mental illness, and other conditions (Hu et al., 2021; Yang et al., 2020; Zhang et al., 2019). A study involving 129 patients diagnosed with Post-Stroke Depression (PSD) demonstrated a significant correlation between increased SII levels on admission and PSD (Mazza et al., 2020).

Numerous studies have constructed ASD risk prediction models combining some demographic and clinical data (Hagos et al., 2024; Liang et al., 2022; Nosanov et al., 2022). Nevertheless, most of the screening processes of parameters are based on univariate and multivariate analyses, which have some limitations in dealing with multicollinearity between variables (Wang et al., 2022). Least Absolute Shrinkage and Selection Operator (LASSO) is a data-mining technique that adds a penalty function to ordinary multiple linear regression, continuously shrinking coefficients to achieve a parsimonious model thereby avoiding the risk of over-fitting and compensating for the shortcomings of traditional logistic regression in variable screening (Tibshirani, 1996). In this study, we firstly combined logistic regression with the LASSO method to develop a visual prediction model for post-traumatic ASD as an exploratory study.

## Materials & Methods

### Participants

Source population: a prospective clinical cohort study involving adult trauma patients was performed from the trauma center of Affiliated Hospital of Zunyi Medical University. The inclusion criteria were as follows: (a) age between 18 and 75 years old; (b) patients were able to cooperate with the study assessments within 2–3 days after hospital admission regardless of whether they had experienced loss of consciousness or coma at the time of injury; (c) trauma patients with blood tests after admission. Exclusion criteria encompassed: (a) presence of other psychiatric comorbidities, including a prolonged history of alcoholism or drug dependence (chronic alcohol misuse and illicit drug use can mimic acute stress symptoms (agitation, insomnia, autonomic hyper-arousal)), making it difficult to ascribe ASD criteria accurately); (b) patients with severe disorders of consciousness (*e.g.*, severe craniocerebral injury, severe organic diseases); (c) patients diagnosed with infectious and immune diseases.

Sampling method: consecutive recruitment by two professional researchers who screened the trauma center electronic register, and no randomisation sampling was used.

**Figure 1:**
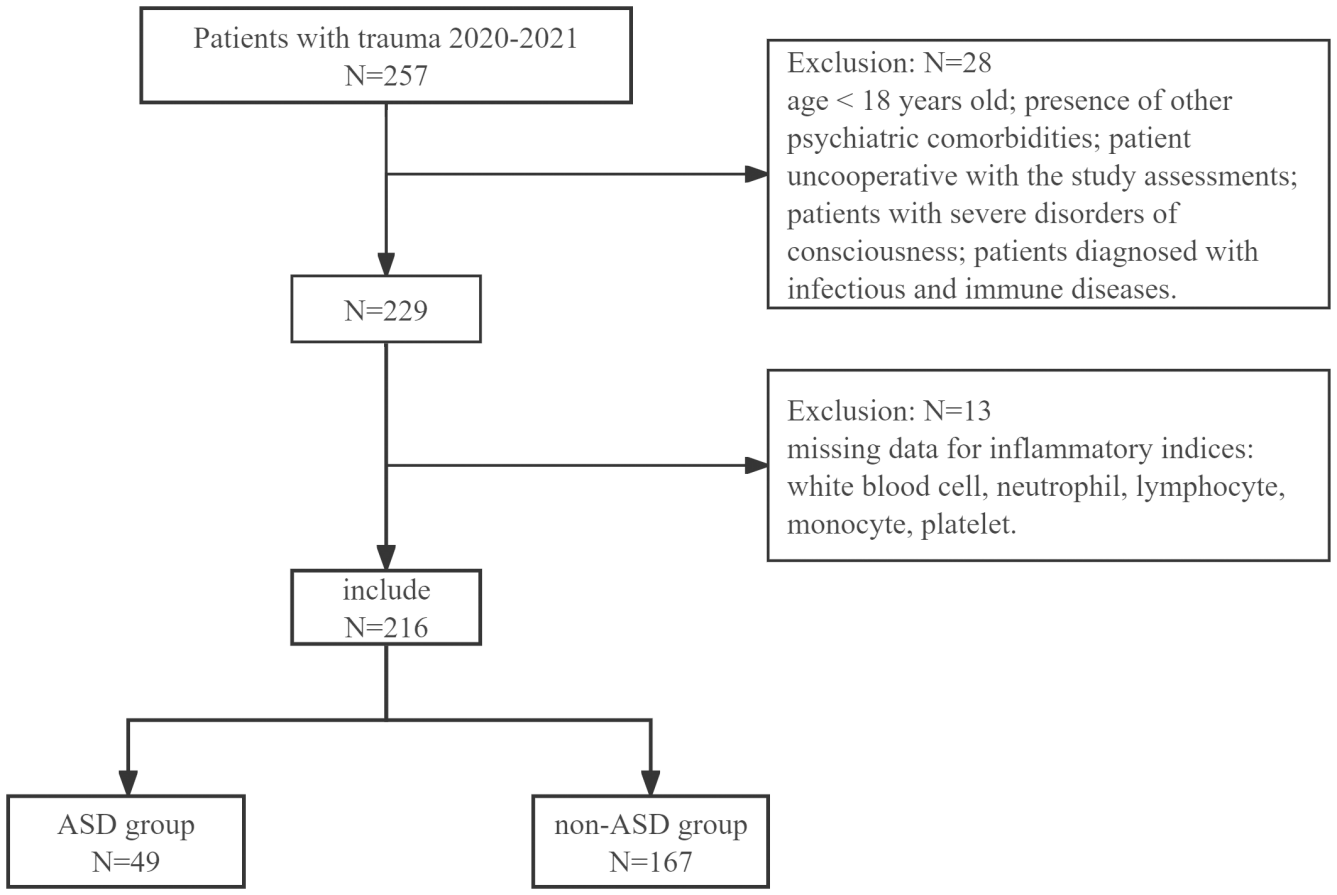
Flowchart of study participation’s selection.

Sample size: based on a literature review, the pooled prevalence (P) of ASD after traumatic events was 29.40% (95% CI [19.50–41.80]), with standard normal distribution (Z_α/2_) = 1.96 and absolute precision tolerable margin of error (D) = ±6% (Given the significant variability reported in ASD-related research, we therefore used the median value) (Geoffrion et al., 2022). According to the sample size estimation formula: N = (Z_α/2_)^2^ × P × (1−P)/*D*^2^ = 1.96^2^  ×  0.294  × (1−0.294)/0.06^2^, the sample size was 221. Considering that this study involved follow-up, we added a 10% allowance for loss to follow-up, and the final calculation indicated that the study should include no fewer than 244 participants. Post-traumatic psychological initial assessment was taken on day 2 after admission, followed by 1–2 additional measurements at approximately 7-day intervals (the exact number of assessments varied owing to differences in length of stay or loss to follow-up). Between September 2020 and August 2021, a total of 257 trauma patients were initially enrolled. Following the application of specific inclusion and exclusion criteria, the final sample comprised 216 patients (See Fig. 1). This research received approval from the Institutional Review Board of Zunyi Medical University (No. ZMU-IRB [2019]1-001), and written informed consent was obtained from all participants or their legal guardians.

### Data collection tools

Pain and injury levels were assessed using the Visual Analogue Scale (VAS), Abbreviated Injury Scale (AIS) and Injury Severity Score (ISS). The Acute Stress Disorder Scale (ASDS) is based on the DSM-4 diagnostic criteria and consists of 19 items, including four symptom groups: dissociation (5), re-experience (4), avoidance (4), and hypervigilance (6). The scale consists of a Likert five-point scale, where each item is rated on a scale of 1 to 5. A score of 1 is “no” and a score of 5 is “always”. The total score ranges from 19 to 95, and the higher the score, the more severe the patient’s ASD symptoms. The ASDS has been shown to possess good sensitivity (95%) and specificity (83%) in a previous study (Bryant, Moulds & Guthrie, 2000). In this study, the ASDS showed high internal reliability (Cronbach α = 0.9). The specific data collection points are detailed in Table 1. Variables collected for further analysis among trauma patients encompassed gender, age (years), smoking status, alcohol use, work or life pressure, trauma history, operation history, cause of trauma, site of trauma, coma after injury, fear after injury, psychological burden, critical condition, sleep quality, limb activity, ISS, VAS, Length of Stay (LOS), hormones, complications.

### Blood analysis and detection

Venous blood was drawn between 07:00 and 09:00 on the first full calendar day after hospital admission. Samples were processed and analyzed using automated blood analyzers and automated chemical analyzers. Various parameters including white blood cell count, lymphocyte count, monocyte count, neutrophil count, and platelet count were obtained. Inflammatory response indexes were computed as follows: NLR, neutrophil count/lymphocyte count; PLR, platelet count/lymphocyte count; MLR, monocyte count /lymphocyte count; SII, (platelet count × neutrophil count)/lymphocyte count. The specific data collection points are detailed in Table 1. Dividing continuous variables such as the NLR into tertiles is a common grouping method (Huang et al., 2024; Li et al., 2024; Xu et al., 2023), which can more intuitively demonstrate the dose–response relationship between variable levels and outcomes. This approach is particularly useful when the non-linear relationship between indicators and study outcomes remains unclear, as tertile-based grouping can reduce bias associated with subjective categorization. Therefore, we categorized NLR, PLR, SII, and other indicators into low, medium, and high groups.

**Table 1:** Time-line of data collection.

Data	Collection time
Demographics & injury characteristics	Recorded within 1 h after arrival at the trauma center
ISS	Calculated by the trauma registrar within 24 h after admission.
VAS	2 days after admission
ASD assessment	Initial post-traumatic psychological assessment was taken on day 2 after admission, followed by 1–2 additional assessments at approximately 7-day intervals.
Venous blood for inflammatory indices: White blood cell, Neutrophil, Lymphocyte, Monocyte, Platelet	drawn at 07:00–09:00 on the first complete calendar day after admission

**Notes.**

ASD Acute Stress Disorder ISS Injury Severity Score VAS Visual Analogue Scale

### Statistical analysis

Statistical analysis was conducted using SPSS (Version 29.0, IBM Corp., Armonk, NY, USA) and R software (Version 4.3.2; R Foundation for Statistical Computing, Vienna, Austria). The continuous variables were described by mean and standard deviation/average rank. The t test/analysis of variance was utilized for inter-group comparisons of normally distributed data, while the non-parametric rank sum test was applied for non-normally distributed data. Categorical data was described as frequency and percentage (%), and the comparison between groups used Chi-square tests/rank sum test. In LASSO regression, a penalty-tuning parameter (lambda) is constructed to shrink some regression coefficients and yield a parsimonious model. Most researchers currently use cross-validation to determine lambda (Obuchi & Kabashima, 2016). In this study, fivefold cross-validation was employed: the data were randomly split into five parts, four folds served as the training set and the remaining fold as the validation set to test the model derived from the training set. This procedure was repeated five times so that each fold served once as the validation set. Following previous study recommendation (Waldmann et al., 2013), lambda1se at the left dotted line was selected as the optimal penalty-tuning parameter (See Fig. 1). Variables selected from LASSO regression were included in multivariable logistic regression to develop the prediction model. The discriminatory capacity of the model was assessed by determining the Area Under the Curve (AUC). Youden’s index combines sensitivity (Se) and specificity (Sp) to summarize a test’s overall ability to distinguish patients from non-patients. The Youden index ranges from 0 to 1, with higher values indicating greater overall validity. Youden’s index is computed as follows: Youden’s index Se + Sp −1. It is commonly used to identify the optimal cut-off point that simultaneously maximizes both sensitivity and specificity. The bootstrapping method (resampling = 500) was used to perform the internal validation (Collins et al., 2015) and the model correction concordance index (C-index) and its 95% CI were calculated after repeated sampling 500 times. Hosmer-Lemeshow test and Decision Curve Analysis (DCA) were used to evaluate the calibration and clinical usefulness of the model, respectively.

## Results

In this study, a total of 216 trauma patients were enrolled, with an average age of 46.90 ± 13.61 years old. Based on the diagnostic criteria for ASD, patients were divided into the ASD group (49 cases) and the control group (167 cases). In terms of general demographic characteristics, patients in the ASD group differed significantly from those in the control group in terms of work or life pressure (*P* = 0.002), trauma history (*P* = 0.047), cause of trauma (*P* < 0.001), trauma site (*P* < 0.001), coma (*P* < 0.001), fear (*P* = 0.002), psychological burden (*P* < 0.001) (Table 2). However, there were no statistical differences between the two groups regarding gender, age, alcohol use, smoking status, and operation history. Regarding clinical characteristics, significant differences were observed between the ASD and control groups in terms of the critical condition (*P* = 0.012), sleep quality (*P* < 0.001), limb activity (*P* = 0.003), ISS (*P* < 0.001), VAS (*P* < 0.001), hormones (*P* = 0.001), and complications (*P* < 0.001). The median hospital LOS in the ASD group was 13 days, which was higher than that in the control group (*P* < 0.001). Regarding blood indicators, the ASD group exhibited higher levels compared to the control group in NLR (*P* = 0.001), PLR (*P* = 0.003), MLR (*P* = 0.034) and SII (*P* = 0.001) (Table 3).

**Table 2:** General demographic characteristics of the ASD and non-ASD groups in trauma.

Variables	ASD (*N* = 49)	non-ASD (*N* = 167)	*P* -value
Gender (Male), n (%)^a^	28 (57.1)	111 (66.5)	0.231
Age (years)^b^	45.80 ± 12.16	47.23 ± 14.02	0.518
Smoking status (Yes), n (%)^a^	20 (40.8)	81 (48.5)	0.343
Alcohol use (Yes), n (%)^a^	12 (24.5)	59 (35.3)	0.156
Work or life pressure (Yes), n (%)^a^	42 (85.7)	103 (61.7)	0.002
Trauma history (Yes), n (%)^a^	6 (12.2)	43 (25.8)	0.047
Operation history (Yes), n (%)^a^	17 (34.7)	68 (40.7)	0.448
Cause of trauma, n (%)^a^			<0.001
Traffic accident	14 (28.6)	16 (9.6)	
Fall from height	6 (12.2)	9 (5.4)	
Other	29 (59.2)	142 (85.0)	
Site of trauma, n (%)^a^			<0.001
Head and neck	9 (18.4)	10 (6.0)	
Trunk	20 (40.8)	35 (21.0)	
Limbs	20 (40.8)	122 (73.1)	
Coma (Yes), n (%)^a^	20 (40.8)	17 (10.2)	<0.001
Fear after injury (Have), n (%)^a^	34 (69.4)	74 (44.3)	0.002
Psychological burden (Have), n (%)^a^	44 (89.8)	87 (52.1)	<0.001

**Notes.**

ASD Acute Stress Disorder

a All data were reported as as frequency and percentage (%) using Chi-square tests.

b All data were reported as mean (standard deviation) using two independent samples *t*-test.

**Table 3:** Clinical characteristics of the ASD and non-ASD groups in trauma.

Variables	ASD group (*N* = 49)	non-ASD (*N* = 167)	*P* -value
Critical condition (Yes), n (%)^a^	10 (20.4)	13 (7.6)	0.012
Sleep quality (Poor), n (%)^a^	44 (89.8)	105 (62.9)	<0.001
Limb activity (Severe limitation), n (%)^a^	31 (63.3)	65 (38.9)	0.003
ISS (>16 points), n (%)^a^	17 (34.7)	14 (8.4)	<0.001
VAS, n (%)^a^			<0.001
≤3 points	3 (6.1)	62 (37.1)	
4–6 points	28 (57.1)	90 (53.9)	
7–10 points	18 (36.7)	15 (9.0)	
LOS^c^	136.68	100.23	<0.001
Hormones (Yes), n (%)^a^	17 (34.7)	24 (14.4)	0.001
Complications (Have), n (%)^a^	25 (51.0)	41 (24.6)	<0.001
White blood cell (×10^9^/ L)^c^	116.67	106.10	0.298
Neutrophil (×10^9^/ L)^c^	123.08	104.22	0.063
Lymphocyte (×10^9^/ L)^c^	86.51	114.95	0.005
Monocyte (×10^9^/ L)^c^	105.27	109.45	0.680
Platelet (×10^9^/ L)^c^	114.18	106.83	0.469
NLR^c^	133.84	101.07	0.001
PLR^c^	131.77	101.67	0.003
MLR^c^	125.12	103.62	0.034
SII^c^	134.24	100.95	0.001

**Notes.**

ASD Acute Stress Disorder ISS Injury Severity Score LOS Length of Stay VAS Visual Analogue Scale NLR Neutrophil to Lymphocyte Ratio PLR Platelet to Lymphocyte Ratio MLR Monocyte to Lymphocyte Ratio SII Systemic Immune-Inflammation Index

a All data were reported as as frequency and percentage (%) using Chi-square tests.

c All data were reported as average rank using the Mann–Whitney U test.

**Figure 2:**
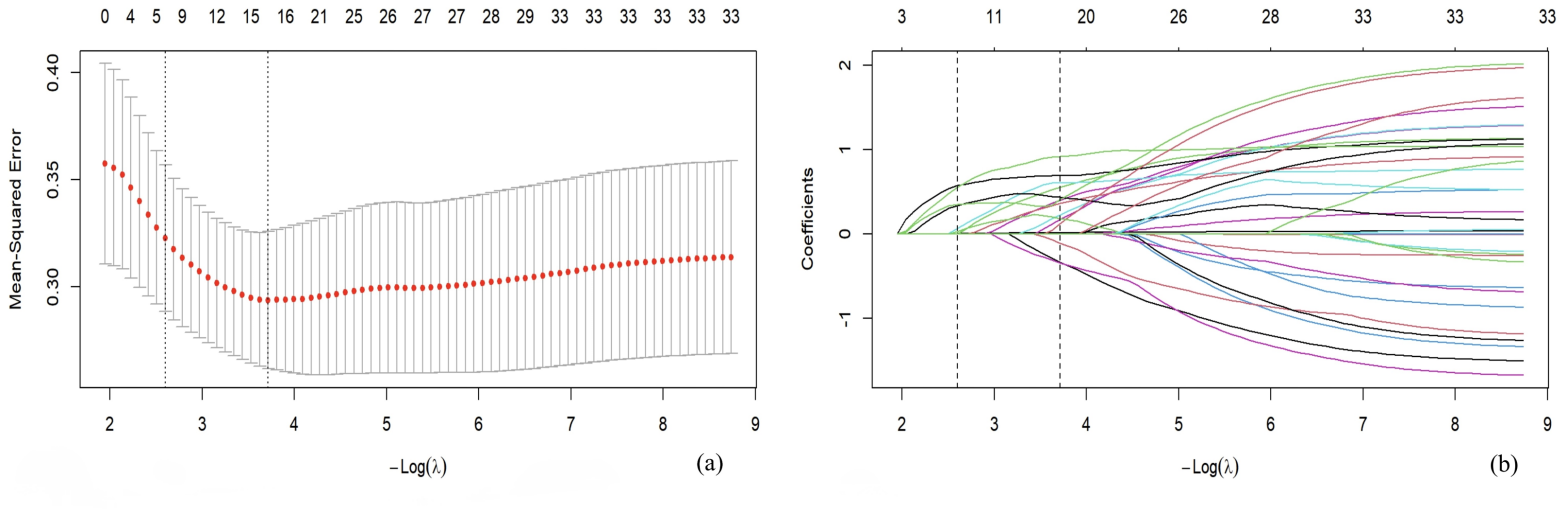
Variables selection using the LASSO regression analysis with fivefold cross-validation. (A) adjustment parameter (lambda) selection of mean-squared error in the LASSO regression based on the lambda. min at the right dotted line (−log(lambda.min) = 3. 99) and the lambda.1se at the left dotted line [−log(lambda.1se) = 2.60). (B) LASSO regression filter variable path process diagram. In this study, lambda value corresponding to the lambda.1se was selected, and seven variables were finally included in the multivariable logistic regression analysis. LASSO, Least Absolute Shrinkage and Selection Operator.

**Figure 3:**
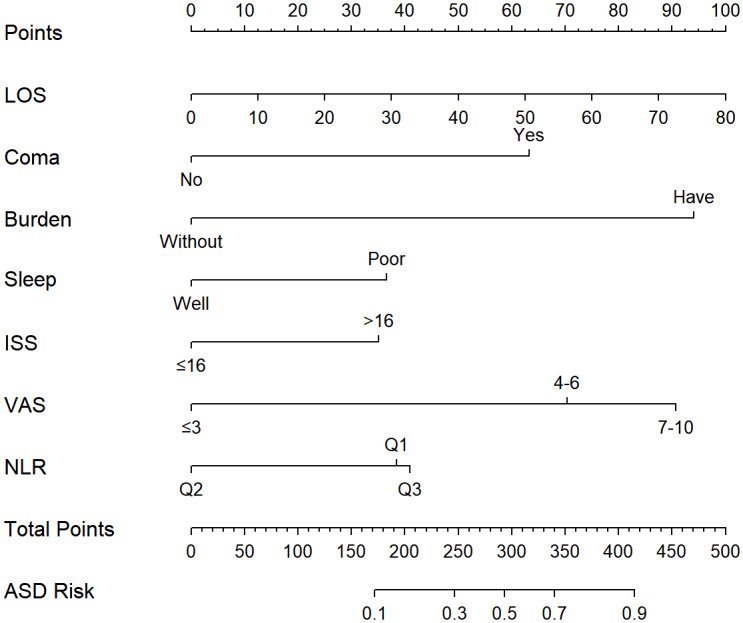
Nomogram for predicting the risk of ASD. At the top of the nomogram, the “Points” represents the scoring criteria for each predictor. The final score was obtained by taking the sum of all scores and corresponding to “Total Points” at the bottom of the graph. Finally, the predicted probability of ASD for the trauma patient was obtained by comparing with the “ASD risk” scale at the bottom. ASD, Acute Stress Disorder; LOS, Length of Stay; ISS, Injury Severity Score; VAS, Visual Analogue Scale; NLR, Neutrophil to Lymphocyte Ratio. Q1:<3.59, Q2:3.59−6.73, Q3: >6.73.

Variables were selected based on fivefold cross-validation calculated by LASSO regression analysis (Fig. 2). Finally, there were a total of seven variables (including LOS, coma after injury, psychological burden, sleep quality, ISS, VAS and NLR) included in the multivariable logistic regression analysis. Coma after injury (Odds Ratio, OR = 3.13 (95% CI [1.17–8.37])) and psychological burden (OR = 5.47 (95% CI [1.97–18.70])) were independent risk factors for ASD (Table 4). The nomogram, as a convenient, personalized tool was built to predicted the probability of ASD in Fig. 3.

**Table 4:** Multivariable logistic regression analysis of influencing factors of the traumatic ASD.

Variables	B	SE	z value	*P-* value	OR (95% CI)
LOS	0.02	0.02	1.11	0.266	1.02 (0.99–1.07)
Coma after injury (Yes)	1.14	0.50	2.29	0.022	3.13 (1.17–8.37)
Psychological burden (Have)	1.70	0.56	3.02	0.003	5.47 (1.97–18.70)
Sleep quality (Poor)	0.66	0.61	1.08	0.282	1.94 (0.61–7.02)
ISS>16	0.63	0.56	1.12	0.261	1.88 (0.62–5.72)
VAS				0.136	
≤3 points (Ref.)^*^					1.00
4–6 points	1.27	0.72	1.76	0.079	3.56 (0.96–17.73)
7–10 points	1.64	0.83	1.98	0.048	5.14 (1.10–30.31)
NLR				0.282	
Q1 (Ref.)					1.00
Q2	−0.69	0.53	−1.31	0.192	0.50 (0.17–1.41)
Q3	0.05	0.53	0.09	0.931	1.05 (0.36–2.98)

**Notes.**

* (Ref.), As a reference group; ASD, Acute Stress Disorder; LOS, Length of Stay; ISS, Injury Severity Score; VAS, Visual Analogue Scale; NLR, Neutrophil to Lymphocyte Ratio; B, Estimate; SE, Standard Error; Q, Quartile; Q1:<3.59, Q2:3.59-6.73, Q3: >6.73; OR, Odds Ratio; CI, Confidence Interval.

**Figure 4:**
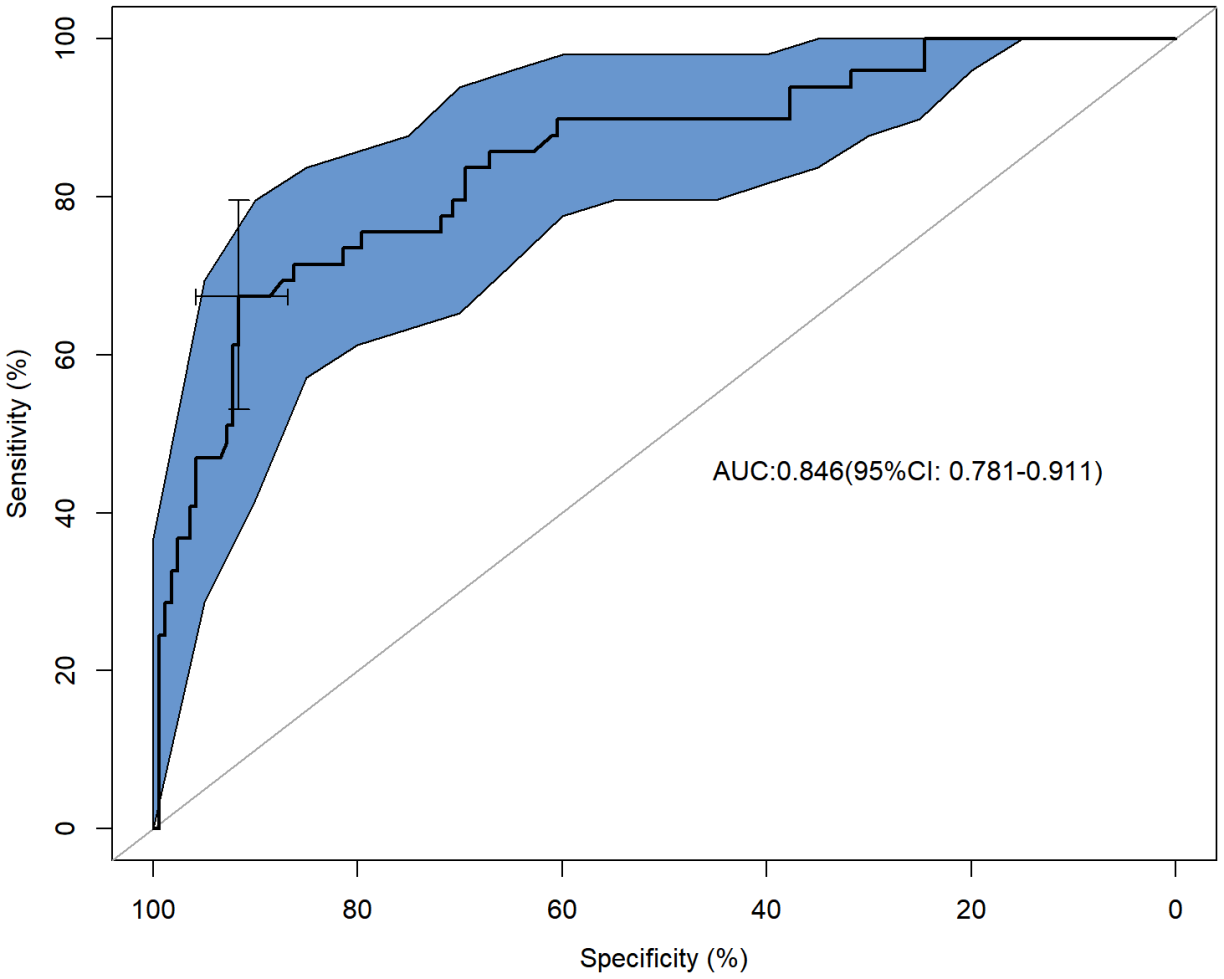
The AUC was determined the discriminatory capacity of the model. The horizontal coordinate was specificity, the vertical coordinate was sensitivity, the shaded area was the 95% confidence interval of the AUC, and the cross coordinate was the optimal diagnostic cut-off point at which the Youden’s index was maximized. AUC, Area Under the Curve.

The predictive model demonstrated an AUC 0.846 (95% CI [0.781–0.911]), The threshold (0.35) corresponded to the optimal diagnostic cut-off point at which the Youden’s index (0.59) was maximized. The sensitivity was determined to be 67.35%, and specificity was 91.62% (Fig. 4). Internal validation of the predictive model was conducted using the bootstrap method (resampling = 500), yielding a C-index of 0.845 (95% CI [0.783–0.911]).

**Figure 5:**
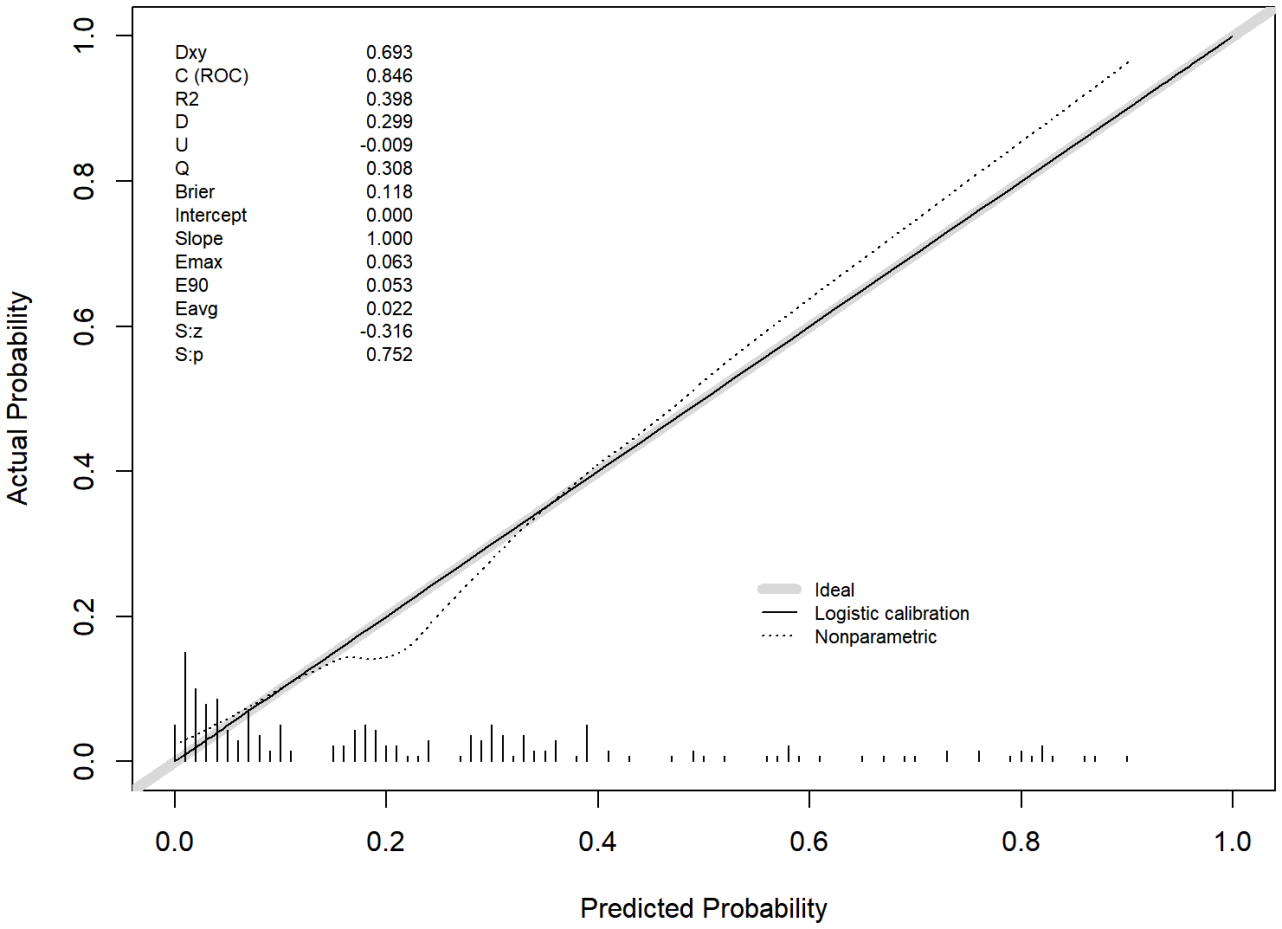
The calibration curve. It showed the degree of consistency between the predicted probability and the observed probability. Dxy: Measures the correlation between predicted and observed values; C (ROC): Area under the ROC curve; *R*^2^: Multiple coefficient of determination; D: Discrimination index; U: Unreliability index; Q: Quality index; Brier: Mean squared error between predictions and observations; Emax: Maximum absolute difference between predicted and observed values; E90: 90th percentile of the absolute differences between predicted and observed values; Eavg: Average difference between predicted and observed values. S:z, S:p: Z-value and *p*-value from the Z-test.

The calibration curve results indicated a strong agreement between the predicted probability and the observed probability. Furthermore, the Hosmer-Lemeshow test yielded a non-significant *P*-value, suggesting that the model exhibited good goodness-of-fit (*χ*^2^ = 11.06, *P* = 0.199) (Fig. 5).

**Figure 6:**
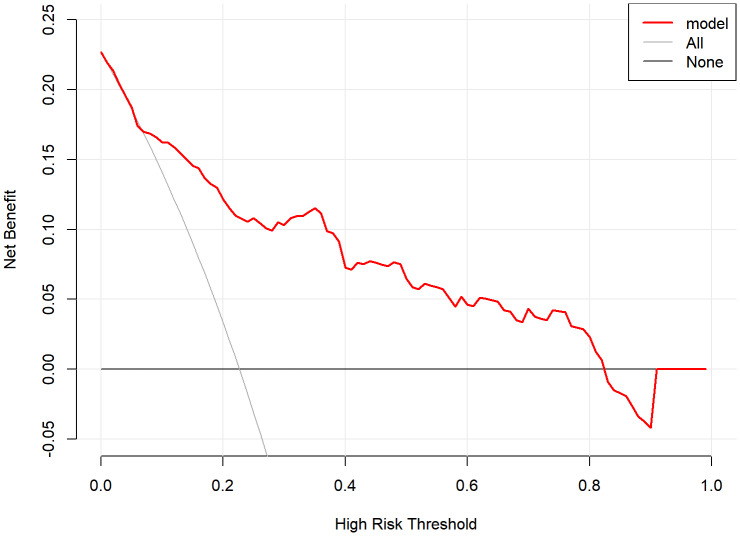
The decision curve. It showed that two reference lines were already displayed: “All” (gray dashed)—intervention-all (prevalence-based strategy); “None” (black dashed)—intervention-none (zero-benefit baseline). The nomogram was compared against two extreme strategies: intervention-all (All) and intervention-none (None). The net benefit was positive between 10% and 83% risk thresholds, outperforming both comparators in this range. ASD, Acute Stress Disorder.

In this study, DCA was employed to assess the net benefit of the predictive model. The DCA revealed that within a risk threshold range of 10% to 83% for ASD, the predictive model yielded positive net benefits compared to both intervention-all and intervention-none strategies (Fig. 6).

## Discussion

ASD represents a critical mental health concern, being the earliest disorder to manifest following injury, and thereby constitutes a significant public health issue (Worku, Tesfaw & Getnet, 2022). Moreover, if ASD remains unrecognized and untreated in its early stages, it may progress to PTSD after one month or more, resulting in heightened physical and psychological repercussions for patients (Haag et al., 2017). While considerable research has focused on identifying risk and protective factors associated with PTSD, the ability to precisely identify ASD early is still limited. To our knowledge, this study represents the first attempt to evaluate a nomogram for ASD in trauma patients as an exploratory study.

In this study, a total of 49 patients were diagnosed with ASD, constituting 22.69% of the total trauma inpatients. The detection rate of ASD observed in our study closely mirrored findings from research conducted in the United States 22.9% (Briere et al., 2017) and Colombia 23.4% (Tamayo-Gómez et al., 2022). However, our study reported a lower detection rate compared to studies conducted in Northwest Ethiopia 45% (Worku, Tesfaw & Getnet, 2022) and New Orleans 62% (Mills, Edmondson & Park, 2007). Additionally, research focused on patients with violence-related injuries documented ASD incidences ranging from 11.70% to 40.60% (Ophuis et al., 2018). A meta-analysis encompassing 66 studies reported a wide range of ASD incidence during hospitalization, spanning from 1% to 37% (Visser et al., 2017). In the study of Northwest Ethiopia, the inclusion criteria contained patients with a personal or family history of psychiatric disorders, as well as those with anxiety disorders. In the study of New Orleans, participants were 132 adults (56% men and 44% women), and the inclusion criteria contained patients with psychiatry history. However, in our study, the exclusion criteria encompassed patients with presence of other psychiatric comorbidities, and male patients accounted for 64% of the cohort. In systematic reviews, the variability in reported ASD incidences across studies may stem from differences in study design, diagnostic criteria, measurement tools, nature of injury, inclusion and exclusion criteria, sample size, and other factors. Therefore, our study may serve as a reference for single-center study investigations based on ASDS according to the DSM-4 diagnostic criteria that exclude patients with age lower than 18 years old and those with a history of psychiatric disorders.

The results of the multivariable logistic regression analysis revealed that coma after injury emerged as an independent predictor of ASD. A research on PTSD following trauma found that there was a threefold risk of PTSD in those with a Glasgow Coma Scale (GCS) lower than 15 (Jemberie et al., 2025). Besides, previous studies consistently have demonstrated that TBI has the potential to be psychologically traumatic and the resulting structural and functional cerebral alterations provide a direct pathobiological substrate for the subsequent emergence of PTSD (Bryant, 2011; Stein et al., 2019). Furthermore, trauma patients burdened with psychological distress were found to be more susceptible to developing ASD. This observation might be attributed to the profound psychological burden experienced by some trauma patients, stemming from the traumatic impact of disfigurement, limb amputation, paraplegia, or even facing life-threatening situations (Huang & Su, 2021). In addition to grappling with the immediate physical consequences of the trauma, these individuals must confront the stark reality of diminished quality of life and loss of livelihood, leading to significant psychological distress and potentially contributing to the development of ASD (Melcer et al., 2019; Momaya et al., 2024).

Pain (VAS (7–10 points), OR = 5.14) might be a potential risk factor for ASD in trauma patients although the overall group effect *P*-value was no statistical significance corroborating findings from research involving 595 elderly patients with osteoporotic fractures (Xiao et al., 2020). Pain, serving as a persistent stressor, often accompanies patients throughout their medical journey, disrupting normal sleep patterns and potentially yielding adverse physiological consequences. Given its significance, pain has been recognized as one of the five vital signs and garners considerable attention in clinical practice. Fuglsang et al. (2002) reported that pain could be a powerful trigger for reexperiencing symptoms, thereby repeatedly reminding patients of the trauma they have gone through. Furthermore, pain can evoke negative emotions in patients and serve as a stressor capable of impacting emotional, cognitive, and physiological processes, thereby predisposing patients to the development of ASD (Wang et al., 2011).

A growing body of evidence supports the involvement of immune and inflammatory pathways in the pathogenesis of mood disorders (Aydin Sunbul et al., 2016; Dionisie et al., 2021; Llorca-Bofí et al., 2021; Miller & Raison, 2016). NLR, PLR, and MLR serve as inexpensive and readily available markers of systemic inflammation (Aydin Sunbul et al., 2016; Cheng et al., 2022; Özdin & Böke, 2019). These inflammatory ratios offer valuable insights beyond other white blood cell parameters or widely used markers such as IL-6, TNF-α, and CRP (Ivković et al., 2016). A study of 256 patients with depression not only revealed significantly elevated levels of NLR in depressed patients but also demonstrated a positive correlation between NLR levels and Hamilton Rating Scale for Depression score (Aydin Sunbul et al., 2016). Consistent with related research findings, our study observed significantly increased levels of NLR, PLR, MLR and SII in patients with ASD compared to the control group (Inanli et al., 2019; Marazziti et al., 2022; Mazza et al., 2018; Zhou, Ma & Wang, 2020; Zhu et al., 2022). The underlying mechanisms might involve recurrent stress-induced damage to the blood–brain barrier, facilitating the penetration of peripheral cells directly into the brain (Aydin Sunbul et al., 2016; Sayed et al., 2020). Upon migration to the brain, peripheral inflammatory cells, including neutrophils, monocytes, and lymphocytes, could exacerbate brain injury and neuronal damage by releasing pro-inflammatory cytokines, neutrophil extracellular traps (NETs), and generating reactive oxygen species and reactive nitrogen species (Cheng et al., 2022). Additionally, pro-inflammatory cytokines might influence psychiatric symptoms by disrupting monoamine neurotransmitter synthesis, affecting glutaminergic signaling, and altering neurogenesis and synaptic plasticity (Cheng et al., 2022; Kim, Amidfar & Won, 2019).

Numerous studies have constructed ASD risk prediction models based on univariate and multivariate analyses, which have some limitations in multicollinearity. In this study, we combined logistic regression with the LASSO method to develop a visual prediction model. This nomogram demonstrated excellent discriminatory ability and showed good effects in calibration. But, the decision-curve indicates net benefit between 10% and 83% predicted-risk thresholds. This broad interval might reflect the small sample size and the limited number of outcome events across risk deciles, leading to wide confidence bands and over-fitting. External validation in a larger cohort will be required to stabilize the threshold range and to identify more precise cut-offs for clinical implementation.

The present study has several limitations. Firstly, our study was a single-center design with a small sample size, and Berkson’s bias and selection bias could not be completely avoided, and our findings could not be generalized to multi-center settings. Secondly, since the questionnaire information came from the self-reports of patients or guardians, which might exist information bias. Thirdly, psychological assessment only was based on ASDS according to the DSM-4 diagnostic criteria, which might influence the detection rate of ASD to some extent. Fourthly, the nomogram included only hemogram-based markers, limiting its biological diversity and generalizability. Finally, this model only based on internal validation should be regarded as exploratory and is not ready for direct clinical decision-making. In addition, we will plan to conduct a multi-center external validation in an independent cohort in the future study.

## Conclusion

In this study, a nomogram for predicting ASD among trauma patients was constructed, which incorporated seven variables based on the demographic and clinical characteristics of trauma patients. This prediction model showed better performance in discriminatory ability and calibration. But, in view of the limited sample size, this model should be regarded as exploratory; its clinical utility and stability require external validation in a larger, multi-center cohort before any consideration of routine adoption.

##  Supplemental Information

10.7717/peerj.21131/supp-1 Supplemental Information 1 Raw data

10.7717/peerj.21131/supp-2 Supplemental Information 2 Variable assignment

## Additional Information and Declarations

### Data Availability

The following information was supplied regarding data availability: The raw data are available in the Supplemental Files.
